# Context-specific effects of NOX4 inactivation in acute myeloid leukemia (AML)

**DOI:** 10.1007/s00432-022-03986-3

**Published:** 2022-03-29

**Authors:** Muhammed Burak Demircan, Tina M. Schnoeder, Peter C. Mgbecheta, Katrin Schröder, Frank-D. Böhmer, Florian H. Heidel

**Affiliations:** 1 Innere Medizin II, Hämatologie und Onkologie, Jena University Hospital, Jena, Germany; 2 Leibniz Institute on Aging, Fritz Lipmann Institute, Jena, Germany; 3 Institute of Molecular Cell Biology, CMB, Jena University Hospital, Jena, Germany; 4 Molecular Biotechnology and Gene Therapy, Paul-Ehrlich-Institut, Langen, Hessen Germany; 5 Innere Medizin C, Universitätsmedizin Greifswald, Greifswald, Germany; 6 Institute for Cardiovascular Physiology, Goethe University, Frankfurt am Main, Germany

**Keywords:** NADPH oxidases (NOX), *Nox4*, Acute myeloid leukemia (AML), FLT3-ITD, Reactive oxygen species (ROS), Oxidative stress, CRISPR-Cas9

## Abstract

**Purpose:**

Oxidative stress has been linked to initiation and progression of cancer and recent studies have indicated a potential translational role regarding modulation of ROS in various cancers, including acute myeloid leukemia (AML). Detailed understanding of the complex machinery regulating ROS including its producer elements in cancer is required to define potential translational therapeutic use. Based on previous studies in acute myeloid leukemia (AML) models, we considered NADPH oxidase (NOX) family members, specifically NOX4 as a potential target in AML.

**Methods:**

Pharmacologic inhibition and genetic inactivation of NOX4 in murine and human models of AML were used to understand its functional role. For genetic inactivation, CRISPR-Cas9 technology was used in human AML cell lines in vitro and genetically engineered knockout mice for Nox4 were used for deletion of Nox4 in hematopoietic cells via *Mx1*-Cre recombinase activation.

**Results:**

Pharmacologic NOX inhibitors and CRISPR-Cas9-mediated inactivation of *NOX4* and *p22-phox* (an essential NOX component) decreased proliferative capacity and cell competition in FLT3-ITD-positive human AML cells. In contrast, conditional deletion of *Nox4* enhanced the myeloproliferative phenotype of an FLT3-ITD induced knock-in mouse model. Finally, *Nox4* inactivation in normal hematopoietic stem and progenitor cells (HSPCs) caused a minor reduction in HSC numbers and reconstitution capacity.

**Conclusion:**

The role of NOX4 in myeloid malignancies appears highly context-dependent and its inactivation results in either enhancing or inhibitory effects. Therefore, targeting NOX4 in FLT3-ITD positive myeloid malignancies requires additional pre-clinical assessment.

**Supplementary Information:**

The online version contains supplementary material available at 10.1007/s00432-022-03986-3.

## Introduction

Acute myeloid leukemia (AML) is a heterogeneous disease of the hematopoietic system characterized by abnormal proliferation and accumulation of immature cells. Clonal evolution and acquisition of additional mutations drive leukemic transformation of myeloid progenitors. Leukemic cells arising from the malignant clone have lost the ability for differentiation and are characterized by reduced apoptosis (Chan and Majeti 2013). With improved understanding of the underlying biology, novel treatment options have emerged for patients with AML (Kantarjian et al. 2021). Of note, development of novel targeted treatment options requires the careful assessment of the molecular target and putative functional differences regarding the cellular context or underlying genetic background (Cucchi et al. 2021).

NADPH oxidases (NOXs) are transmembrane proteins that generate reactive oxygen species (ROS) as their sole function (Altenhöfer et al. 2012). NOX4 and NOX2 are expressed in hematopoietic cancers and contribute to cellular transformation by increasing ROS-levels and genomic instability (Naughton et al. 2009; Reddy et al. 2011; Stanicka et al. 2015). Recently, our group observed that genetic inactivation of *Nox4* attenuated FLT3-ITD-driven leukemic transformation. Consistently, pharmacologic targeting of NOX4 decreased proliferative capacity of FLT3-ITD transformed cells (Jayavelu et al. 2016). In contrast, recent studies found tumor-promoting effects of *Nox4* deficiency (Helfinger et al. 2021). Therefore, we sought to validate and confirm the therapeutic potential of NOX4 as a therapeutic target in AML.

## Materials and methods

### Cell lines, cell proliferation and apoptosis

Murine 32D-FLT3-ITD were provided by Prof. J. Duyster, Freiburg, Germany and Dr. R. Grundler, Munich, Germany). Leukemia cell lines were purchased from DSMZ (Braunschweig, Germany). Cells were cultured according to standard protocols and tested negative for mycoplasma. Cell viability was measured using Cell Titer-Blue reagent (Promega, Madison, WI, USA) according to the manufacturers’ instructions. For proliferation assays, the number of cells was counted with a hemocytometer. Apoptosis was measured by flow cytometry using Annexin V/SYTOX Blue staining.

### Use of pharmacologic inhibitors

The following inhibitors were used for in vitro studies: ROS inhibitors: GKT137831 (Selleckchem, Houston, TX, USA) and GSK2795039 (Hycultec GmbH, Beutelsbach, Germany).

### Genetic inactivation by CRISPR/Cas9

Genetic inactivation by CRISPR/Cas9 was performed as previously described (Perner et al. 2021; Schnoeder et al. 2022) unless otherwise stated. Guide RNAs were designed using the Broad GPP tool (Doench, Nat Biotechnology 2014). For cloning of sgRNA sequences, the improved-scaffold-pU6-sgRNA-EF1Alpha-PURO-T2A-RFP (ipUSEPR) vector system (Uckelmann et al. 2018), with puromycin resistance and RFP selection marker was used. Genetic inactivation by CRISPR/Cas9 was performed as published before (Jayavelu et al. 2020). sgRNA sequences are provided in the Supplementary Materials. For negative selection competition assays, transduced cells were mixed with non-transduced cells at 9:1 RFP^−^/RFP^+^ ratio for applying selection pressure. The percentage of RFP^+^ was monitored by flow cytometry.

### Genomic *NOX4* knockout validation

PCR on genomic DNA was used to confirm *NOX4* knockout in human AML cell lines. Primer pairs are listed in the Supplementary Materials.

### Protein extraction and immunoblotting

Western Blotting was performed according to standard protocols as previously published (Heidel et al. 2006; Schnöder et al. 2015). Cell lines and whole bone marrow cells were lysed as described previously. Two different antibodies against NOX4 were provided by J. M. Doroshow (Bethesda, MD, USA) (Meitzler et al. 2017) and A. Shah (King’s College London, London, UK) (Anilkumar et al. 2008). All antibodies are indicated in the Supplementary Materials.

### Animal experiments

All mice were kept under pathogen-free conditions in the accredited Animal Research facility of the University Hospital Jena. The animal experiments were approved by the Landesverwaltungsamt Thüringen (animal protocol number 02-035/16). Experimental mice were generated by crossing *Nox4*^flox/flox^ mice (Schröder et al. 2012) with Mx1-Cre transgenic mice (B6.Cg-Tg(*Mx1*-Cre)1Cgn/J: Jackson Laboratory, Bar Harbor, USA) and *Flt3*^ITD/ITD^ knock-in mice (Lee et al. 2007). Mx1-Cre-recombinase was activated by intraperitoneal injections of 100 µg low-molecular-weight poly-I-poly-C (LMW-pIpC, GE-Healthcare) on alternating days as indicated.

### Competitive bone marrow (BM) transplantation and analysis of steady state hematopoiesis

Competitive bone marrow transplantation and analysis of steady state hematopoiesis were performed as previously published (Mohr et al. 2018). *Nox4*^flox/flox^ *Mx1*-Cre + mice and *Nox4*^flox/flox^ *Mx1*-Cre- or *Nox4*^wt/wt^ *Mx1*-Cre + control mice were used as donors.

Excision control regarding *Nox4* was performed on genomic DNA isolated from WBM cells. PCR primers included Nox4-forward, CCAAGCTTCCGATTCCCATTCTC and Nox4-reverse, GTCCTC-CAATCATGAAAGTGAAGC). An alternative forward primer was used to detect the 509 bp unexcised loxP-flanked allele (Nox4-forward-alt: AGAATGAAAAGCTAGGCGTCCTTGG).

### Flow cytometry and antibody staining

Immunophenotyping of normal and leukemic cell compartments and of leukemic PB and BM was performed as described before (Heidel et al. 2012, 2013). Antibodies are provided in Supplementary Methods. Flow cytometry was performed on a FACS Canto II cytometer (Becton Dickinson, Heidelberg, Germany).

## Results

**Fig. 1:**
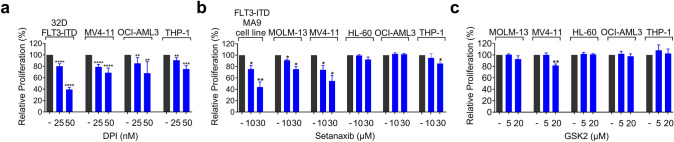
Effect of NOX inhibitors on the growth of AML cells. **a, b** The proliferation/viability of the leukemic cells was measured by Cell Titer Blue assay. Upon 72 h of treatment, the fluorescent signals, which are directly proportional to the number of viable cells, were measured and normalized to the fluorescent signal of the DMSO control group. Three independent experiments (technical triplicates) were conducted. **a** DPI treatment (25 nM and 50 nM) **b** Setanaxib treatment (10 µM and 30 µM) and **c** GSK2795039 treatment (5 µM and 20 µM) (*P < 0.05, **P < 0.01, ***P < 0.001, ****P < 0.0001 by two-tailed *t-*test)

**Fig. 2:**
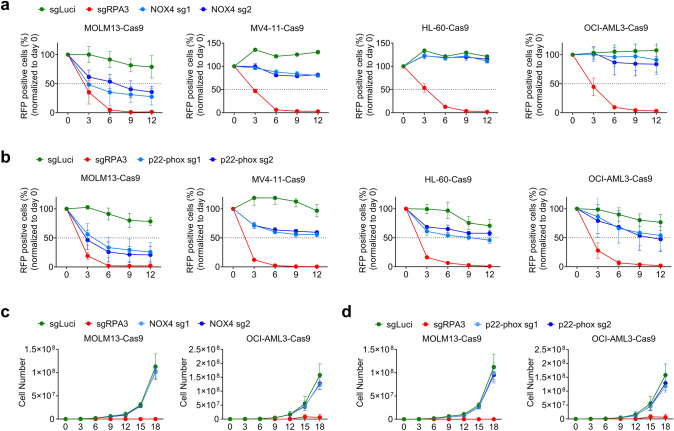
Effect of genetic inactivation of NOXs on cell competition and proliferation of human AML cells. **a, b** CRISPR-Cas9 cell competition assay in MOLM13, OCI-AML3, MV4-11 and HL-60 cells after (**a**) NOX4 or (**b**) p22-phox deletion using 2 different sgRNAs compared to knockout of RPA3 (positive control) or a sgRNA (sgLuci) targeting luciferase (negative control) through 12 days (*x*-axis) after starting the competition assay. sgRNA targeted transduced (RFP^+^) cells were mixed with their non-transduced counterparts (RFP^−^) at a 1:10 ratio. **c, d** Proliferation was assessed by cell counting with trypan blue exclusion in (**c)** NOX4 or (**d)** p22-phox knockout in MOLM13 and OCI-AML3 cells over 18 days (*x*-axis). No significant difference was found between sgLuci and NOX4 or p22-phox targeted groups using two-tailed *t*-test

First, we sought to define the potential of pharmacological NOX-inhibition in more detail using murine and human cell models of AML. Treatment with the rather unselective NOX inhibitor DPI reduced proliferative capacity of leukemia cell lines (Fig. 1a). However, as DPI is not suitable for in vivo treatment and known to inhibit some other enzymes like eNOS, and xanthine oxidase (Altenhöfer et al. 2015), we decided to test the more selective NOX4/1 inhibitor setanaxib and the NOX2 inhibitor GSK2795039. While murine and human FLT3-ITD-positive AML cell lines showed higher sensitivity to setanaxib (Fig. 1b), only MV4-11 cells were inhibited in growth by GSK2795039 (Fig. 1c). Given the potential off-target effects of NOX-inhibitors (Augsburger et al. 2019), we aimed to validate the sensitivity of FLT3-ITD cell lines by genetic inactivation of NOX-enzymes. Human AML cell lines with stable expression of Cas9 were generated as described before (Jayavelu et al. 2020; Perner et al. 2021; Schnoeder et al. 2022). To assess the functional importance of NOX4 (*NOX4*) and of the essential NOX-component p22-phox (*CYBA*), we used a CRISPR–Cas9-mediated gene-editing and negative-selection strategy. The genetic deletion was confirmed by Western blotting, and gPCR (Supplementary Fig. S1 and S2). Cells expressing different NOX4 single guide RNAs (sgRNAs) were (partially) outcompeted by non-transduced cells in FLT3-ITD positive cell lines (MOLM13, MV4-11), while non-FLT3-mutated cell lines (OCI-AML3, HL-60) appeared less sensitive (Fig. 2a). In contrast, at least partial dropout was observed in all cell lines with p22-phox targeting sgRNAs (Fig. 2b). The more pronounced phenotype of p22-phox targeting compared to NOX4 targeting may reflect the essential function of p22-phox for several NOX-enzymes (NOX1-4). However, we observed rather subtle effects of NOX4 and p22-phox deletion on leukemia cell proliferation or apoptosis compared to cell competition and negative selection assays (Fig. 2c, d and Supplementary Fig. S3). Taken together, these in vitro results suggest that NOX4 is relevant under conditions of leukemia cell competition in FLT3-mutated cell lines.

ROS balance and NOX activity in cells in vivo are also regulated by cell-extrinsic factors and neighboring niche cells (Tarafdar and Pula 2018). Also, recent publications provide evidence for the functional relevance of *Nox2* in normal hematopoiesis and for leukemogenesis in mice (Adane et al. 2019). Therefore, we sought to confirm the above effects of *Nox4* on FLT3-mutated myeloid disease in vivo. In brief, conditional *Nox4*^fl/fl^ mice (Schröder et al. 2012) were crossed with an *Mx1*-Cre-recombinase (B6.Cg-Tg(*Mx1*-Cre)1Cgn/J, received from The Jackson Laboratory, Bar Harbor, USA) and a conventional *Flt3-*ITD knock-in mouse strain (Lee et al. 2007).

**Fig. 3:**
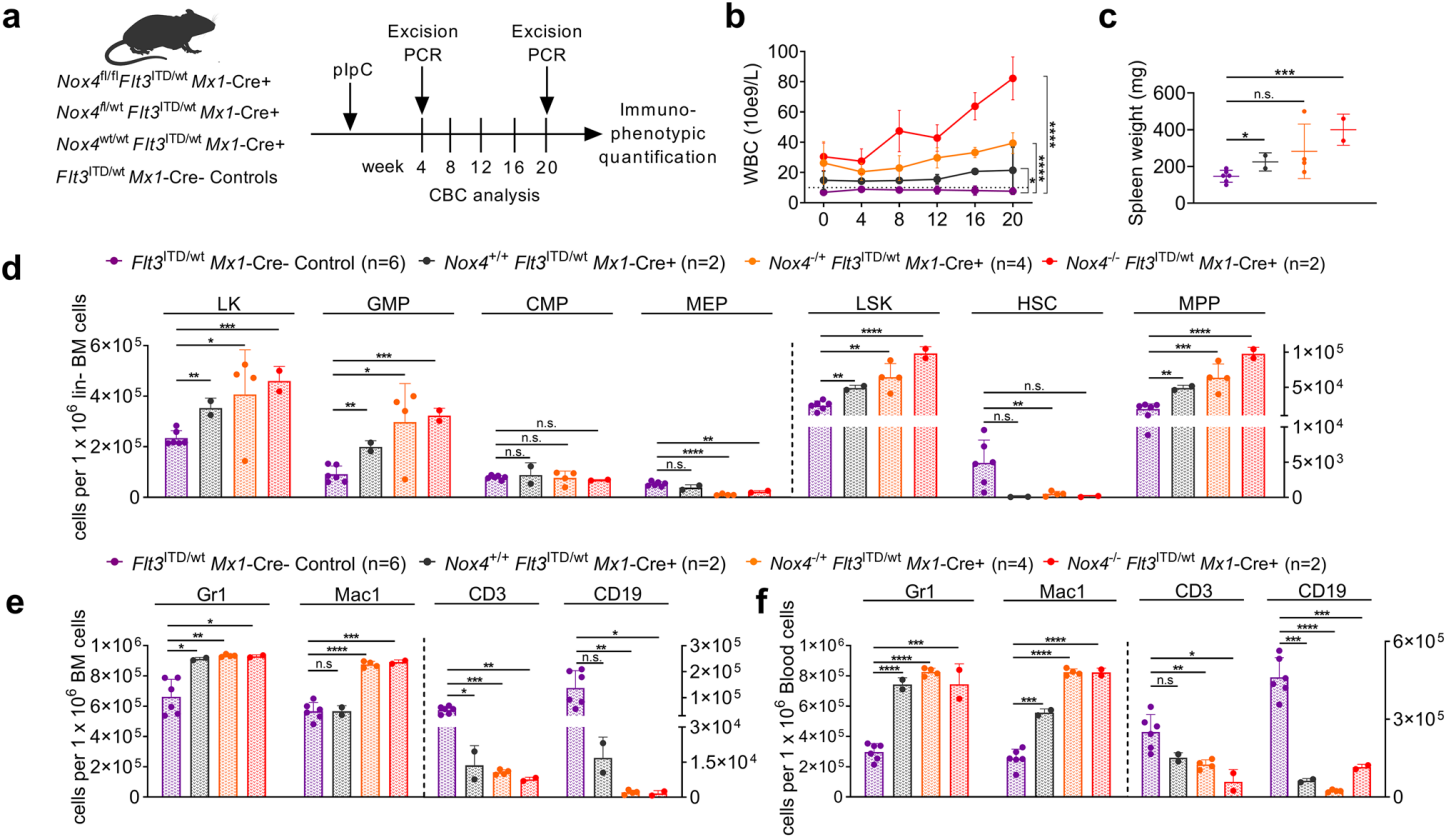
Role of *Nox4* in the context of FLT3-ITD-driven myeloproliferative disease. **a** Experimental protocol for assessing the impact of *Nox4* deletion on steady-state hematopoiesis in *Flt3*^ITD/wt^ *Mx1*-Cre + mice. **b** WBCs after conditional deletion of *Nox4* through 20 weeks. **c** Spleen weights of *Nox4* wt or knockout *Flt3*^ITD/wt^ *Mx1*-Cre + mice. **d** Immunophenotypic quantification of stem- and progenitor cell abundance, specifically of L^−^K^+^, L^−^S^+^K^+^, HSC (CD48^−^CD150^+^L^−^S^+^K^+^), MPP (CD48^+^CD150^+^L-S^+^K^+^ or CD48^+^CD150^−^L^−^S^+^K^+^ or CD48^−^CD150^−^L^−^S^+^K^+^), GMP (CD34^high^FcgR^high^L^−^S^−^K^+^), CMP (CD34^high^FcgR^low^L^−^S^−^K^+^), and MEP (CD34^low^FcgR^low^L^−^S^−^K^+^). **e, f** Immunophenotypic quantification of mature myeloid (Gr-1^+^ or Mac1^+^), B-lymphoid (CD19^+^), and T-lymphoid (CD3^+^) cells in **e** BM and **f** blood. Error bars indicate the standard deviation. (*P < 0.05, **P < 0.01, ***P < 0.001, ****P < 0.0001 by two-tailed *t*-test)

**Fig. 4:**
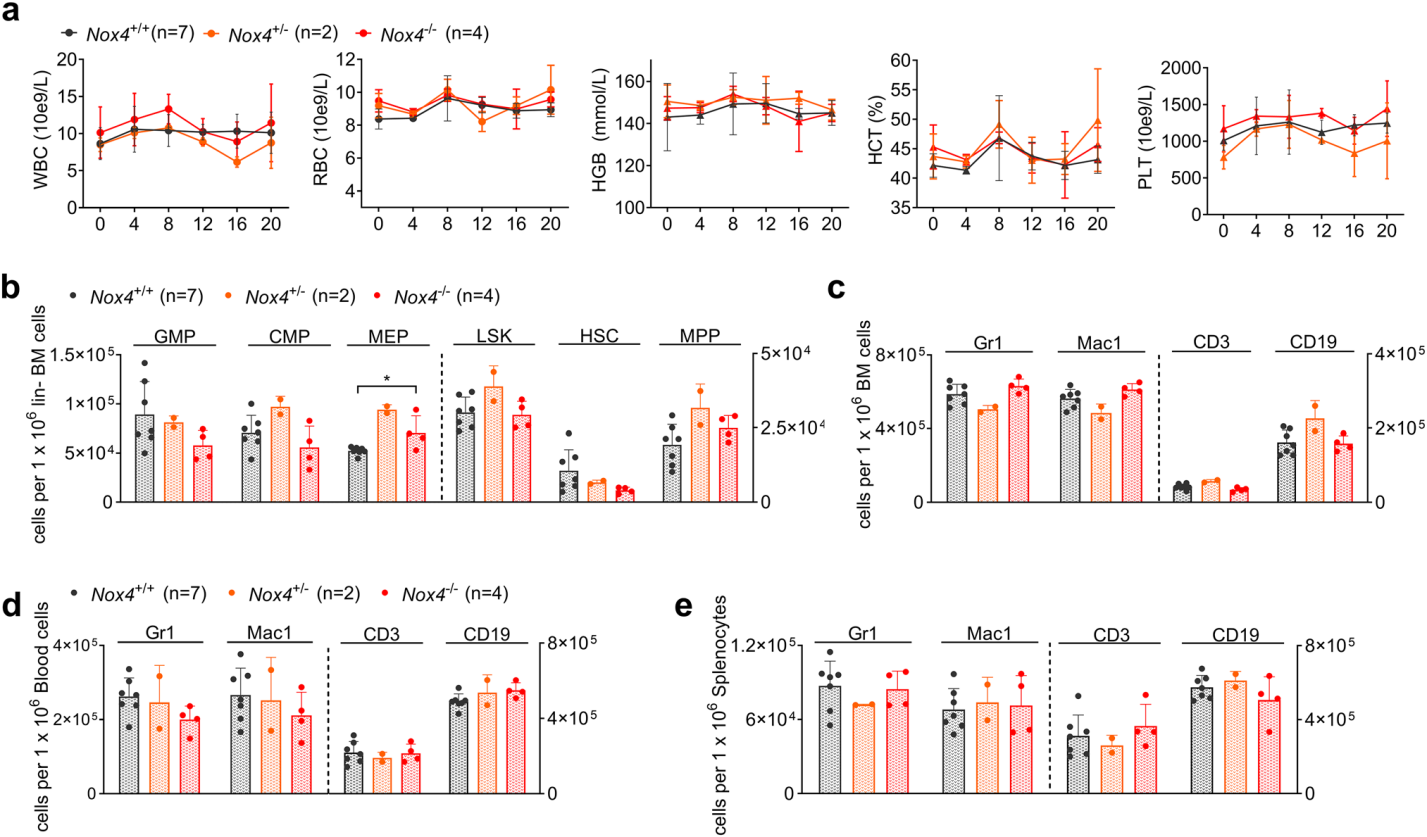
Role of *Nox4* in normal steady-state hematopoiesis. **a** Peripheral blood counts; white blood count (WBC), red blood count (RBC), hematocrit (HCT) level, hemoglobin (HGB) level, and platelet count (PLT) after conditional deletion of *Nox4* during normal steady-state hematopoiesis through 20 weeks. **b** Immunophenotypic quantification of HSPCs as indicated in Fig. 3d. **c–e** Immunophenotypic quantification of mature myeloid (Gr-1^+^ or Mac1^+^), B-lymphoid (CD19^+^), and T-lymphoid (CD3^+^) cells in **c** BM, **d** peripheral blood and **e** spleen. Error bars indicate the standard deviation. (*P < 0.05, by two-tailed *t*-test)

**Fig. 5:**
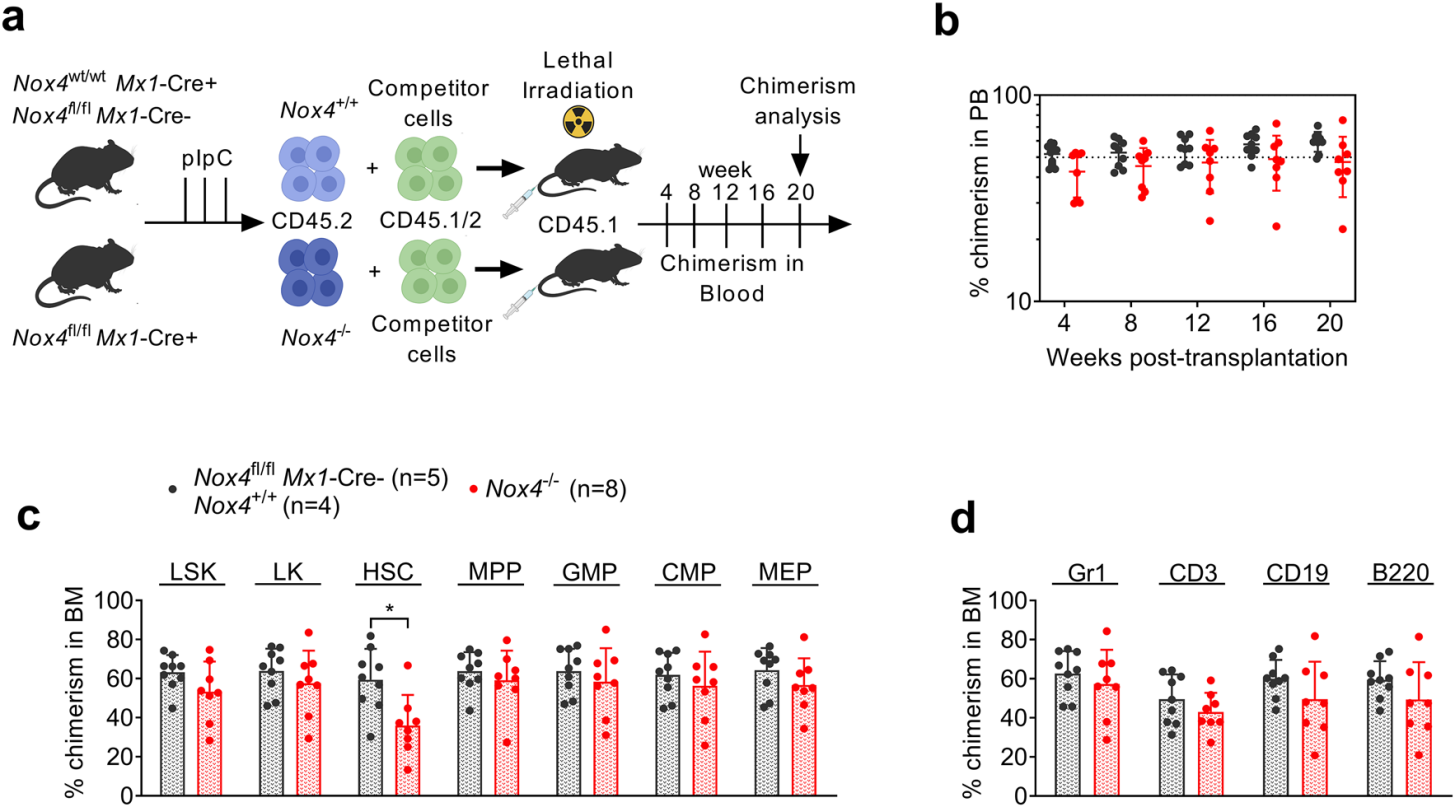
Impact of *Nox4* deletion on the reconstituting capability of HSPCs. **a** Experimental protocol for assessing the impact of *Nox4* deletion on repopulation capacity of HPSCs. Competitive repopulation assay was performed by the competition of *Nox4*^−/−^ or *Nox4*^+/+^ donor cells against WT competitor cells. **b** Peripheral blood chimerism of primary recipient mice. *P* value = 0.045 for the difference on day 20. **c** Chimerism of immature BM compartments: L-K + , L-S + K + , HSC (CD34^−/low^L^−^S^+^K^+^), MPP (CD34^+^L^−^S^+^K^+^), GMP (CD34^high^FcgR^high^L^−^S^−^K^+^), CMP (CD34^high^FcgR^low^L^−^S^−^K^+^), and MEP (CD34^low^FcgR^low^L^−^S^−^K^+^). *P* value = 0.011 for the difference in HSC compartment. **d** Chimerism of myeloid (Gr1^+^) and lymphoid (CD3^+^, CD19^+^, B220^+^) linages in BM. Two independent cohorts; *Nox4*^−/−^ (*n* = 8), controls *Nox4*^+/+^ (*n* = 4) and *Nox4*^fl/fl^ *Mx1*-Cre + (*n* = 5), mean ± SD. Statistical analyses were performed using a two-tailed Mann–Whitney test

This model allows functional assessment of disease development in vivo and its dependency on *Nox4* without being dependent on bone marrow transplantation and irradiation. *Mx1*-Cre recombinase was induced by administration of pIpC in *Nox4*^fl/fl^ *Flt3*^ITD/wt^ *Mx1*-Cre + , *Nox4*^fl/wt^ *Flt3*^ITD/wt^ *Mx1*-Cre + , or *Nox4*^wt/wt^ *Flt3*^ITD/wt^ *Mx1*-Cre + mice and *Mx1*-Cre- littermate controls (Fig. 3a**).** Excision of the *Nox4* gene was confirmed by PCR in PB cells at week 4 (Supplementary Fig. S4b, c). As expected, *Nox4*^wt/wt^ *Flt3*^ITD/wt^ *Mx1*-Cre + mice showed elevated white blood count (WBC) compared to *Flt3*^ITD/wt^ *Mx1*-Cre- controls. Unexpectedly, *Nox4* deletion in *Flt3*^ITD/wt^ *Mx1*-Cre + background induced a further increase in leukocytosis in a gene-dose-dependent manner (Fig. 3b). Consistent with this finding, splenomegaly was more pronounced in *Nox4* deficient animals, when investigated at week 20 (Fig. 3c). Immunophenotypic analysis of stem and progenitor cell compartments in the BM revealed that *Nox4* deletion resulted in increased cell numbers of LKs, LSKs, MPPs, and GMPs in *Flt3*^ITD/wt^ *Mx1*-Cre + mice. Conversely, the number of MEPs was decreased in *Nox4* deficient mice, indicating a lineage bias depending on the presence of *Nox4*. As described before (Lee et al. 2007; Li et al. 2008), HSC numbers appeared reduced in FLT3-mutated animals when compared to wildtype controls (Fig. 3d). Immunophenotypic analysis of mature cell compartments in BM, PB, and spleen revealed an expanded myeloid compartment with increased Mac1 expression in *Nox4* knockout mice and conversely reduced abundance of lymphoid cells (Fig. 3e, f and Supplementary Fig. S4a). Counter-selection of partially excised clones could be excluded by PCR of peripheral blood cells at week 20 (Supplementary Fig. S4d, e). Taken together, these results suggest for the first time that *Nox4* deletion may promote the myeloproliferative phenotype in a FLT3-ITD-driven mouse model. To assess for a functional impact of *Nox4* inactivation on normal HSCs, we investigated steady-state hematopoiesis after conditional deletion of *Nox4* (Supplementary Fig. S5). Following activation of *Mx1*-Cre by pIpC injections into *Nox4*^fl/fl^ *Mx1*-Cre + , *Nox4*^fl/wt^ *Mx1*-Cre + , *Nox4*^wt/wt^ *Mx1*-Cre + or Cre-negative mice, we found no significant differences in peripheral blood counts (Fig. 4a) and immunophenotypic analysis of myeloid or lymphoid blood compartments in peripheral blood, BM, and spleen (Fig. 4c–e). The total number of hematopoietic stem and progenitor cells (HSPCs) was not altered by *Nox4* deletion except for a subtle increase in MEP numbers (Fig. 4b). To test for the function of *Nox4* deficient HSPCs, we used the most stringent assay to assess for hematopoietic stem and progenitor cell function (Purton and Scadden 2007), which is transplantation into irradiated recipient hosts (Fig. 5a). When *Nox4*^−/−^ whole bone marrow cells and *Nox4*^+/+^ controls were transplanted into primary recipient hosts in a competitive manner (ratio of 1:1), we found no loss or gain of function in *Nox4*-deficient cells. *Nox4* knockout cells (Supplementary Fig. S6a, b) competed against wildtype controls as indicated by stable PB chimerism over 16 weeks and showed a mild decrease on week 20 (Fig. 5b). Likewise, no difference was observed in chimerism of total BM or immunophenotypic abundance of immature or mature compartments at week 20 except for rather decreased numbers of *Nox4*^−/−^ cells in the CD34^−^ LSK cell population (Fig. 5c, d). This finding is in line with a previous study, highlighting a decrease in repopulation capacity of *Nox4* knockout HSCs in secondary recipient hosts (Prieto-Bermejo et al. 2021). Taken together, inactivation of *Nox4* does not enhance normal HSPC function in vivo but may result in decreased abundance and function of long-term HSCs.

## Discussion

For the first time, our findings provide evidence that *Nox4* deletion may enhance an FLT3-ITD induced myeloproliferative phenotype in vivo. This phenotype is in contrast to the in vitro data of previous reports: genetic and pharmacologic targeting of NOX4 decreased the growth of leukemic cells in vitro (Jayavelu et al. 2016; Naughton et al. 2009; Reddy et al. 2011)*,* which is also consistent with our genetic targeting approach in human AML. Using transplantation models of FLT3-ITD driven cells into irradiated recipient hosts, genetic inactivation of *Nox4* by RNAi had resulted in prolonged survival. Of note, these models had been performed using murine AML cell lines or primary murine cells harboring more than one oncogene (MLL-AF9 and FLT3-ITD).

As indicated above, a recent study investigated the role of *Nox4* in a different cancer model and described a tumor suppressor role of *Nox4*. In this study, *Nox4* deletion enhanced cancerogen-induced tumor formation in murine models of carcinoma and sarcoma (Helfinger et al. 2021). The phenotype observed here suggests that the role of *Nox4* in leukemia is highly context dependent. Targeting *Nox4* may therefore result in leukemia inhibitory or promoting effects and underlines the necessary caution in developing NOX4-modulating drugs. Moreover, suitable compounds that may specifically target NOX4 are still lacking. Setanaxib (GKT137831), a drug currently developed in advanced clinical trials, was recently proposed as a NOX1/4 specific inhibitor. However, its specificity regarding NOX enzymes has recently been questioned (Augsburger et al. 2019; Dao et al. 2020). Detailed pre-clinical genetic and pharmacologic studies of NOX4 modulation are clearly required before defining it as a bona fide target in leukemia therapy.

## Supplementary Information

Below is the link to the electronic supplementary material.

Supplementary file1 (DOCX 10460 KB)

## References

[CR1] Adane B, Ye H, Khan N, Pei S, Minhajuddin M, Stevens BM, Jones CL, D’Alessandro A, Reisz JA, Zaberezhnyy V (2019) The hematopoietic oxidase NOX2 regulates self-renewal of leukemic stem cells. Cell Rep 27:238–254 30943405 10.1016/j.celrep.2019.03.009 PMC6931909

[CR2] Altenhöfer S, Kleikers PW, Radermacher KA, Scheurer P, Rob Hermans J, Schiffers P, Ho H, Wingler K, Schmidt HH (2012) The NOX toolbox: validating the role of NADPH oxidases in physiology and disease. Cell Mol Life Sci 69:2327–2343 22648375 10.1007/s00018-012-1010-9 PMC3383958

[CR3] Altenhöfer S, Radermacher KA, Kleikers PW, Wingler K, Schmidt HH (2015) Evolution of NADPH oxidase inhibitors: selectivity and mechanisms for target engagement. Antioxid Redox Signal 23:406–427 24383718 10.1089/ars.2013.5814 PMC4543484

[CR4] Anilkumar N, Weber R, Zhang M, Brewer A, Shah AM (2008) Nox4 and nox2 NADPH oxidases mediate distinct cellular redox signaling responses to agonist stimulation. Arterioscler Thromb Vasc Biol 28:1347–1354 18467643 10.1161/ATVBAHA.108.164277

[CR5] Augsburger F, Filippova A, Rasti D, Seredenina T, Lam M, Maghzal G, Mahiout Z, Jansen-Dürr P, Knaus UG, Doroshow J (2019) Pharmacological characterization of the seven human NOX isoforms and their inhibitors. Redox Biol 26:101272 31330481 10.1016/j.redox.2019.101272 PMC6658998

[CR6] Chan SM, Majeti R (2013) Role of DNMT3A, TET2, and IDH1/2 mutations in pre-leukemic stem cells in acute myeloid leukemia. Int J Hematol 98:648–657 23949914 10.1007/s12185-013-1407-8 PMC5542003

[CR7] Cucchi DG, Polak TB, Ossenkoppele GJ, Groot UD, Carin A, Cloos J, Zweegman S, Janssen JJ (2021) Two decades of targeted therapies in acute myeloid leukemia. Leukemia 35:651–660 33589753 10.1038/s41375-021-01164-x

[CR8] Dao VT-V, Elbatreek MH, Altenhöfer S, Casas AI, Pachado MP, Neullens CT, Knaus UG, Schmidt HH (2020) Isoform-selective NADPH oxidase inhibitor panel for pharmacological target validation. Free Radical Biol Med 148:60–69 31883469 10.1016/j.freeradbiomed.2019.12.038

[CR9] Heidel F, Solem FK, Breitenbuecher F, Lipka DB, Kasper S, Thiede M, Brandts C, Serve H, Roesel J, Giles F (2006) Clinical resistance to the kinase inhibitor PKC412 in acute myeloid leukemia by mutation of Asn-676 in the FLT3 tyrosine kinase domain. Blood 107:293–300 16150941 10.1182/blood-2005-06-2469

[CR10] Heidel FH, Bullinger L, Feng Z, Wang Z, Neff TA, Stein L, Kalaitzidis D, Lane SW, Armstrong SA (2012) Genetic and pharmacologic inhibition of β-catenin targets imatinib-resistant leukemia stem cells in CML. Cell Stem Cell 10:412–424 22482506 10.1016/j.stem.2012.02.017 PMC3339412

[CR11] Heidel FH, Bullinger L, Arreba-Tutusaus P, Wang Z, Gaebel J, Hirt C, Niederwieser D, Lane SW, Döhner K, Vasioukhin V (2013) The cell fate determinant Llgl1 influences HSC fitness and prognosis in AML. J Exp Med 210:15–22 23277453 10.1084/jem.20120596 PMC3549713

[CR12] Helfinger V, Von Gall FF, Henke N, Kunze MM, Schmid T, Rezende F, Heidler J, Wittig I, Radeke HH, Marschall V (2021) Genetic deletion of Nox4 enhances cancerogen-induced formation of solid tumors. Proc Natl Acad Sci 118 10.1073/pnas.2020152118 PMC7980388 33836590

[CR13] Jayavelu A, Müller J, Bauer R, Böhmer S, Lässig J, Cerny-Reiterer S, Sperr W, Valent P, Maurer B, Moriggl R (2016) NOX4-driven ROS formation mediates PTP inactivation and cell transformation in FLT3ITD-positive AML cells. Leukemia 30:473–483 26308771 10.1038/leu.2015.234

[CR14] Jayavelu AK, Schnoder TM, Perner F, Herzog C, Meiler A, Krishnamoorthy G, Huber N, Mohr J, Edelmann-Stephan B, Austin R et al (2020) Splicing factor YBX1 mediates persistence of JAK2-mutated neoplasms. Nature 588:157–163 33239784 10.1038/s41586-020-2968-3

[CR15] Kantarjian H, Kadia T, DiNardo C, Daver N, Borthakur G, Jabbour E, Garcia-Manero G, Konopleva M, Ravandi F (2021) Acute myeloid leukemia: current progress and future directions. Blood Cancer J 11:1–25 33619261 10.1038/s41408-021-00425-3 PMC7900255

[CR16] Lee BH, Tothova Z, Levine RL, Anderson K, Buza-Vidas N, Cullen DE, McDowell EP, Adelsperger J, Fröhling S, Huntly BJ (2007) FLT3 mutations confer enhanced proliferation and survival properties to multipotent progenitors in a murine model of chronic myelomonocytic leukemia. Cancer Cell 12:367–380 17936561 10.1016/j.ccr.2007.08.031 PMC2104473

[CR17] Li L, Piloto O, Nguyen HB, Greenberg K, Takamiya K, Racke F, Huso D, Small D (2008) Knock-in of an internal tandem duplication mutation into murine FLT3 confers myeloproliferative disease in a mouse model. Blood J Am Soc Hematol 111:3849–3858 10.1182/blood-2007-08-109942 PMC2275036 18245664

[CR18] Meitzler JL, Makhlouf HR, Antony S, Wu Y, Butcher D, Jiang G, Juhasz A, Lu J, Dahan I, Jansen-Dürr P (2017) Decoding NADPH oxidase 4 expression in human tumors. Redox Biol 13:182–195 28578276 10.1016/j.redox.2017.05.016 PMC5458090

[CR19] Mohr J, Dash BP, Schnoeder TM, Wolleschak D, Herzog C, Tubio Santamaria N, Weinert S, Godavarthy S, Zanetti C, Naumann M (2018) The cell fate determinant Scribble is required for maintenance of hematopoietic stem cell function. Leukemia 32:1211–1221 29467485 10.1038/s41375-018-0025-0

[CR20] Naughton R, Quiney C, Turner S, Cotter T (2009) Bcr-Abl-mediated redox regulation of the PI3K/AKT pathway. Leukemia 23:1432–1440 19295548 10.1038/leu.2009.49

[CR21] Perner F, Schnoeder TM, Xiong Y, Jayavelu AK, Mashamba N, Santamaria NT, Huber N, Todorova K, Hatton C, Perner B (2021) YBX1 mediates translation of oncogenic transcripts to control cell competition in AML. Leukemia 1–12 10.1038/s41375-021-01393-0 PMC8807392 34465866

[CR22] Prieto-Bermejo R, Romo-González M, Pérez-Fernández A, García-Tuñón I, Sánchez-Martín M, Hernández-Hernández Á (2021) Cyba-deficient mice display an increase in hematopoietic stem cells and an overproduction of immunoglobulins. Haematologica 106:142 31919083 10.3324/haematol.2019.233064 PMC7776239

[CR23] Purton LE, Scadden DT (2007) Limiting factors in murine hematopoietic stem cell assays. Cell Stem Cell 1:263–270 18371361 10.1016/j.stem.2007.08.016

[CR24] Reddy MM, Fernandes MS, Salgia R, Levine RL, Griffin JD, Sattler M (2011) NADPH oxidases regulate cell growth and migration in myeloid cells transformed by oncogenic tyrosine kinases. Leukemia 25:281–289 21072051 10.1038/leu.2010.263 PMC4078661

[CR25] Schnöder T, Arreba-Tutusaus P, Griehl I, Bullinger L, Buschbeck M, Lane S, Döhner K, Plass C, Lipka D, Heidel F (2015) Epo-induced erythroid maturation is dependent on Plcγ1 signaling. Cell Death Differ 22:974–985 25394487 10.1038/cdd.2014.186 PMC4423181

[CR26] Schnoeder TM, Schwarzer A, Jayavelu AK, Hsu C-J, Kirkpatrick J, Döhner K, Perner F, Eifert T, Huber N, Arreba-Tutusaus P (2022) PLCG1 is required for AML1-ETO leukemia stem cell self-renewal. Blood J Am Soc Hematol 139:1080–1097 10.1182/blood.2021012778 PMC8854675 34695195

[CR27] Schröder K, Zhang M, Benkhoff S, Mieth A, Pliquett R, Kosowski J, Kruse C, Luedike P, Michaelis UR, Weissmann N (2012) Nox4 is a protective reactive oxygen species generating vascular NADPH oxidase. Circ Res 110:1217–1225 22456182 10.1161/CIRCRESAHA.112.267054

[CR28] Stanicka J, Russell EG, Woolley JF, Cotter TG (2015) NADPH oxidase-generated hydrogen peroxide induces DNA damage in mutant FLT3-expressing leukemia cells. J Biol Chem 290:9348–9361 25697362 10.1074/jbc.M113.510495 PMC4392242

[CR29] Tarafdar A, Pula G (2018) The role of NADPH oxidases and oxidative stress in neurodegenerative disorders. Int J Mol Sci 19:3824 30513656 10.3390/ijms19123824 PMC6321244

[CR30] Uckelmann HJ, Kim SM, Antonissen NJ, Krivtsov AV, Hatton C, McGeehan GM, Levine RL, Vassiliou GS, Armstrong SA (2018) MLL-Menin inhibition reverses pre-leukemic progenitor self-renewal induced by NPM1 mutations and prevents AML development. Blood 132:546

