# Supplementary material for: Context-specific effects of NOX4 inactivation in acute myeloid leukemia (AML)
Source: J Cancer Res Clin Oncol. 2022 Mar 29;148(8):1983–90. doi: 10.1007/s00432-022-03986-3 (PMC9293823; doi:10.1007/s00432-022-03986-3)
Supplement: Supplementary file 1 — Supplementary file1 (DOCX 10460 KB) [file 432_2022_3986_MOESM1_ESM.docx]

**Context-specific Effects of NOX4 Inactivation in Acute Myeloid Leukemia (AML)**

Muhammed Burak Demircan ^1,2,3,4^, Tina M. Schnoeder ^5^, Peter C. Mgbecheta ^3^, Katrin Schröder ^6^, Frank-D. Böhmer ^3*^ and Florian H. Heidel ^1,2,5 *^

1 Innere Medizin II, Hämatologie und Onkologie, Jena University Hospital, Jena, Germany

2 Leibniz Institute on Aging - Fritz Lipmann Institute, Jena, Germany

3 Institute of Molecular Cell Biology, CMB, Jena University Hospital, Jena, Germany

4 Molecular Biotechnology and Gene Therapy, Paul-Ehrlich-Institut, (Hessen), Germany

5 Innere Medizin C, Universitätsmedizin Greifswald, Greifswald, Germany

6 Institute for Cardiovascular Physiology, Goethe University, Frankfurt am Main, Germany

**Supplementary Figures:**

**Figure S1**. Validation of NOX4 knockout in inducible NOX4 overexpressing HEKT cells by western blot and in AML cells by a PCR-based method. (**a**) Validation of Cas9 (160 kDa) expression by westernblot in the indicated cell lines which were transduced with lentiviral particles encoding Cas9 (lentiCas9-Blast) (**b-c**) After 8 days of puromycin selection of the sgRNA transduced HEKT-NOX4-Cas9 cells, NOX4 (~65 kDa) expression was induced by tetracycline (1 μg/ml) treatment for 24 hours. Subsequently, the cells were lysed, separated by SDS-PAGE and subjected to immunoblotting with (**b**) homemade NOX4 antibody from Dr. J. M. Doroshow lab [1] or (**c**) homemade NOX4 antibody from Prof. A. Shah Lab [2] (**d, e**) PCR was performed using the indicated primers and the genomic DNA from sgRNA transduced HEKT-NOX4-Cas9 cells after 8 days of puromycin selection (**f-k**) Genomic PCR for the human AML cell lines transduced with different sgRNAs targeting NOX4 (sg1, sg2) or a sgRNA control (sgLuci). (**f, g**) MOLM-13-Cas9, (**h, i**) OCI-AML3-Cas9, (**j**) MV4-11-Cas9, and (**k**) HL-60-Cas9.

**Figure S2.** Validation of p22-phox knockout by westernblot in the human AML cell lines: MOLM-13-Cas9, OCI-AML3-Cas9, MV4-11-Cas9, and HL-60-Cas9.

**Figure S3.** (**a**) Proliferation was assessed upon NOX4 knockout in MV4-11-Cas9 and HL-60-Cas9 cells over 18 days (x-axis). (**b-c**) Apoptosis was measured upon NOX4 knockout in (**b**) MOLM-13-Cas9 or OCI-AML3-Cas9 and (**c**) MV4-11-Cas9 or HL-60-Cas9 cells by flow cytometry on day 8 after infection using Annexin V/Sytox Blue staining. (**d**) Apoptosis was measured upon p22-phox knockout in MOLM-13-Cas9 and OCI-AML3-Cas9 cells by flow cytometry on day 8 after infection using Annexin V/Sytox Blue staining. Bar plot shows quantification of the percentages of Annexin V^+^ cells. Statistical analyses were done using two-tailed *t*-test and the groups which showed significant difference compared to sgLuci control were indicated as * P < 0.05, ** P < 0.01.

**Figure S4.** (**a**) Immunophenotypic quantification of mature myeloid (Gr-1^+^ or Mac1^+^), B-lymphoid (CD19^+^), and T-lymphoid (CD3^+^) cells in spleen (*P < 0.05, **P < 0.01, ***P < 0.001, ****P < 0.0001 by two-tailed *t*-test). (**b**) Genomic DNA was isolated from peripheral blood cells of *Nox4*^-/+^ *Flt3*^ITD/wt^ *Mx1*-Cre+ mice (n=3) and *Nox4*^-/-^ *Flt3*^ITD/wt^ *Mx1*-Cre+ mouse (n=1) at week 4. (**c**) Genomic DNA was isolated from peripheral blood cells of *Nox4*^wt/wt^ *Flt3*^ITD/wt^ *Mx1*-Cre- mice (n=2) and *Nox4*^wt/wt^ *Flt3*^ITD/wt^ *Mx1*-Cre+ mice (n=2) at week 4. (**d**) Genomic DNA was isolated from WBM cells of *Nox4*^-/+^ *Flt3*^ITD/wt^ *Mx1*-Cre+ mice (n=3) and *Nox4*^-/-^ *Flt3*^ITD/wt^ *Mx1*-Cre+ mouse (n=1) at week 20. (**e**) Genomic DNA was isolated from WBM cells of *Nox4*^wt/wt^ *Flt3*^ITD/wt^ *Mx1*-Cre- mice (n=2) and *Nox4*^wt/wt^ *Flt3*^ITD/wt^ *Mx1*-Cre+ mice (n=2) at week 20. Band sizes: Floxed allele at 509 bp, WT allele at 394 bp, and Recombined allele at 350 bp.

**
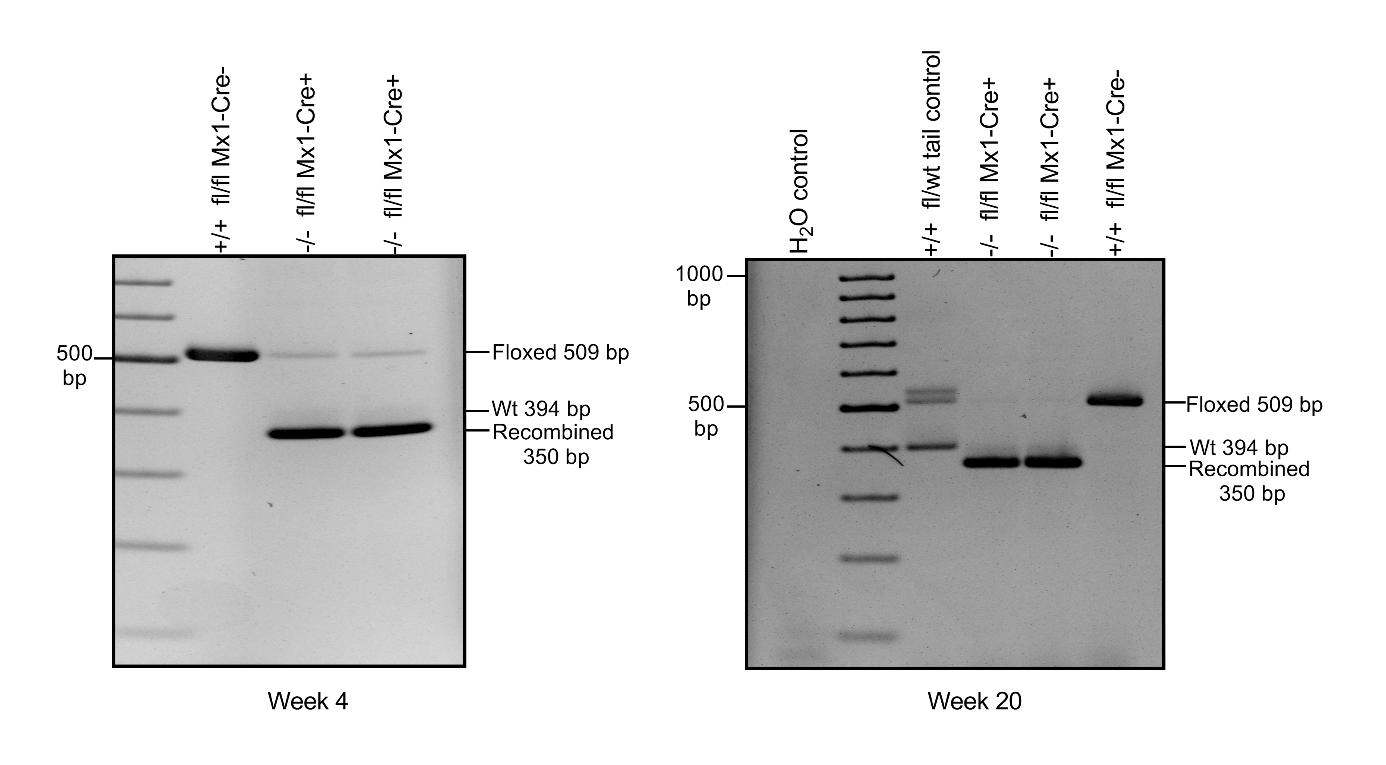
**

**Figure S5.** Confirmation of excision of the conditional knockout mice following genetic inactivation of *Nox4*. Representative gel picture of the PCR products for blood cells at week 4 (left) and WBM cells at week 20 (right). Band sizes: Floxed allele at 509 bp, WT allele at 394 bp, and Recombined allele at 350 bp.


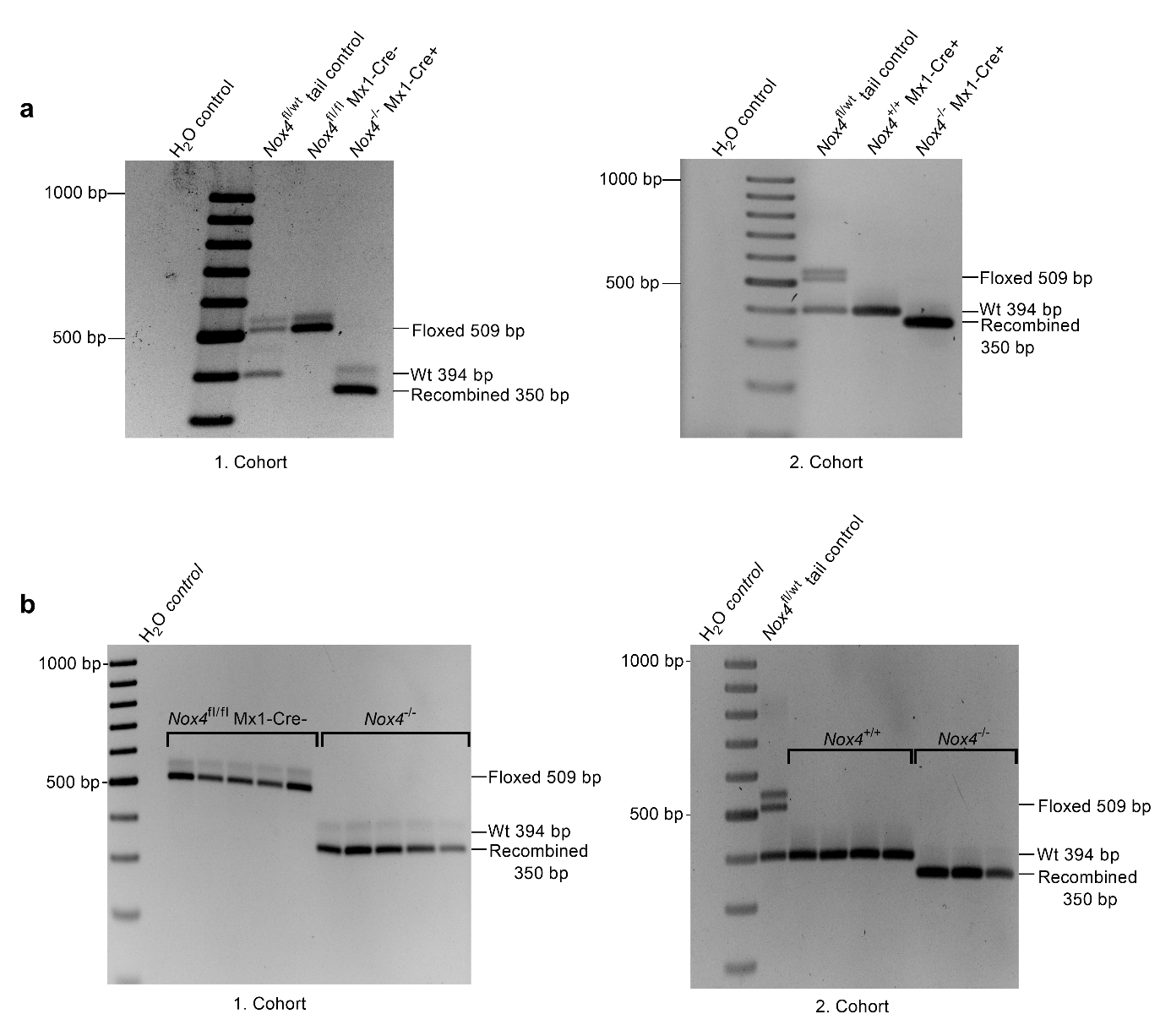


**Figure S6.** (**a**) Competitive repopulation assay excision control PCR on genomic DNA of WBM upon genetic inactivation of *Nox4* in conditional knockout mice. (**b**) PCR on genomic DNA of sorted CD45.2^+^ cells at week 20 following the repopulation assay. No counter-selection of partially excised clones was confirmed. Pictures of two independent cohorts are shown; *Nox4*^-/-^ (n=8), controls (*Nox4*^+/+^ (n=4) and *Nox4*^fl/fl^ *Mx1*-Cre- (n=5)).

**Supplementary Materials:**

**Supplemental Table 1. sgRNA sequences used in this study.**

| **Gene** | **Sequence (5`-3`)** |
| --- | --- |
| *NOX4* sgRNA#1 | GAGGTTAAGAACAGATGCTG |
| *NOX4* sgRNA#2 | TGTTTATAGGACGTCCTCGG |
| p22-phox (*CYBA*) sgRNA#1 | CCCCGCAGTCCTCATCACCG |
| p22-phox (*CYBA*) sgRNA#2 | GACTCACAGGAGATGCAGGA |
| Luciferase sgRNA | GATTCTAAAACGGATTACCA |
| RPA3 sgRNA | GGTTGGAAGAGTAACCGCCA |

**Supplemental Table 2. Primer sequences used in NOX4 knockout validation via RT-PCR.**

| **Target** | **Primer type** | **Primer sequence (5`-3`)** | **Expected band size** |
| --- | --- | --- | --- |
| ***NOX4***  **sgRNA 1** | Control  Forward | GCAGTGCAGAACAGAAAGAAGTCG | Control pair: 485 bp  sgRNA binding-site pair: 243 bp |
|  | sgRNA Binding Site Reverse | CAGTTGAGGTTAAGAACAGATGCTG |  |
|  | Control  Reverse | CTCACAGATCATCTTGGCAGGTG |  |
| ***NOX4***  **sgRNA 2** | Control  Forward | CCGCAGCACCAGCAGAAATG | Control pair: 447 bp  sgRNA binding-site pair: 333 bp |
|  | sgRNA Binding Site Forward | TCAAGACTGTTTATAGGACGTCCTC |  |
|  | Control  Reverse | AGGAGAGTTTAGCTCATAAATACACTG |  |

Supplemental Table 3. Flow cytometry and western blot antibodies used in this study.

| **Antibody** | **Source** | **Identifier** |
| --- | --- | --- |
| APC anti-mouse CD45.2 | Biolegend | Cat #: 109814 |
| PE anti-mouse CD45.1 | Biolegend | Cat #: 110708 |
| APC-Cy7 anti-mouse CD3 | Biolegend | Cat #: 100221 |
| Pacific Blue anti-mouse Gr-1 | Biolegend | Cat #: 108430 |
| PE anti-mouse Gr-1 | Biolegend | Cat #: 108408 |
| FITC anti-mouse CD19 | Biolegend | Cat #: 115524 |
| Pacific Blue anti-mouse CD11b | Biolegend | Cat #: 101224 |
| PE-Cy7 anti-mouse B220 | Biolegend | Cat #: 103221 |
| PE-Cy7 anti-mouse FcgR | Biolegend | Cat #: 101318 |
| APC-Cy7 anti-mouse CD48 | Biolegend | Cat #: 103431 |
| PerCP-Cy5.5 anti-mouse CD150 | Biolegend | Cat #: 115921 |
| PerCP-Cy5.5 anti-mouse Sca-1 | Biolegend | Cat #: 108123 |
| FITC anti-mouse CD34 | BD Biosciences | Cat #: 553733 |
| AF 647 anti-mouse cKit | Biolegend | Cat #: 105818 |
| APC-Cy7 anti-mouse cKit | Biolegend | Cat #: 105826 |
| BV 421 anti-Streptavidin | Biolegend | Cat #: 405226 |
| Biotin anti-mouse TER119 | Biolegend | Cat #: 116204 |
| Biotin anti-mouse CD4 | Biolegend | Cat #: 100404 |
| Biotin anti-mouse CD19 | Biolegend | Cat #: 115503 |
| Biotin anti-mouse Gr-1 | Biolegend | Cat #: 108404 |
| Biotin anti-mouse CD8a | Biolegend | Cat #: 100704 |
| Biotin anti-mouse IL-7Rα | Biolegend | Cat #: 121104 |
| Biotin anti-mouse CD45R/B220 | Biolegend | Cat #: 103203 |
| Biotin anti-mouse CD3ε | Biolegend | Cat #: 100304 |
| Anti-NOX4 (1:500) | received from Dr. J. M. Doroshow, Bethesda, MD, USA (Meitzler et al., 2017) |  |
| Anti-NOX4 (1:1000) | received from Prof. A. Shah Lab King’s College London British Heart Foundation Centre, London, UK (Anilkumar et al., 2008) |  |
| Anti-p22-phox (1:1000) | Bioworld Technology | Cat #: BS60290 |
| Anti-GAPDH (1:5000) | Meridian Life Science | Cat #: H86504M |

**References**

Anilkumar, N., Weber, R., Zhang, M., Brewer, A., and Shah, A.M. (2008). Nox4 and nox2 NADPH oxidases mediate distinct cellular redox signaling responses to agonist stimulation. Arteriosclerosis, thrombosis, and vascular biology *28*, 1347-1354.

Meitzler, J.L., Makhlouf, H.R., Antony, S., Wu, Y., Butcher, D., Jiang, G., Juhasz, A., Lu, J., Dahan, I., and Jansen-Dürr, P. (2017). Decoding NADPH oxidase 4 expression in human tumors. Redox biology *13*, 182-195.
